# Prevalence and profile of users and non-users of anabolic steroids among resistance training practitioners

**DOI:** 10.1186/s12889-019-8004-6

**Published:** 2019-12-09

**Authors:** Ericson PEREIRA, Samuel Jorge MOYSES, Sérgio Aparecido IGNÁCIO, Daniel Komarchewski MENDES, Diego Sgarbi D. A. SILVA, Everdan CARNEIRO, Ana Maria Trindade Grégio HARDY, Edvaldo Antônio Ribeiro ROSA, Patrícia Vida Cassi BETTEGA, Aline Cristina Batista Rodrigues JOHANN

**Affiliations:** 1 Graduate Program, Life Sciences School, Pontifical Catholic University of Parana, Imaculada Conceição, 1155, Prado Velho, Curitiba, PR CEP 8021 Brazil; 2 Department of Pharmacology, College of Pharmacy and Pharmaceutical Sciences, The University of Toledo, 2801 W. Bancroft, Toledo, OH USA

**Keywords:** Anabolic agents, Prevalence, Resistance training, Cross-sectional studies

## Abstract

**Background:**

To verify the prevalence and profile of users and non-users of anabolic steroid (AS) among resistance training practitioners.

**Methods:**

An observational, cross-sectional survey was performed in 100 gyms in Curitiba city, involving 5773 individuals and self-administered questionnaires. The chi-square and z-tests of proportions were used for comparison between the groups (*p* < 0.05).

**Results:**

83.2% did not use, 9.1% formerly used, 3.4% currently used, and 4.3% intended used AS. The prevalence of former or current AS users was 16.9 and 6.5% among men and women, respectively. The prevalence ratios were as follows: 1) 2.6 male users for each woman; 2) 3.3 individuals aged 30–44 years and 2.8 individuals aged 18–29 years for each individual aged over 45 years. Beginners were not interested in using AS, but individuals who had trained longer had higher prevalence of AS use.

**Conclusions:**

The gym environment encouraged the use of AS owing to aesthetic appeal. Thus, suggesting the need for actions to prevent abusive use of AS considering the practitioners profile (practitioners were young, university and single).

## Background

Anabolic steroids (AS) are medications containing synthetic testosterone, the male hormone. These medications may exert anabolic effects related to the growth of and increase in muscle mass, as well as androgenic effects related to male sexual characteristics [1]. Generally, AS are used for treating diseases such as hypogonadism [2] and growth deficits [3]. However, they have become a public health problem with their increased use among athletes and non-athletes for aesthetic reasons [4–6].

Inappropriate use of these medications can result in atherosclerosis, hypertension, cardiac arrhythmias, liver cancer, and prostatic hypertrophy [4, 7], as well as problems such as acne, infertility, and gynecomastia [4]. Injectable AS may also cause pyomyositis [8]. In addition, behavioral problems, such as aggressiveness and mood changes, may be associated with AS use [9].

Global prevalence of AS was estimated to be a meta-analysis in 3.3% [10]. However, this meta-analysis used studies with diverse samples, such as students, university students, resistance training practitioners, and the general public, among others.

In 2001, it was estimated that 0.3% of the adult population in Brazil used AS [11]. In 2005, this number increased to 0.9%, which comprised mostly of men aged between 18 and 34 years [12]. Some studies assessed the prevalence of AS use among resistance training practitioners in gyms from various regions of the country, resulting in a prevalence range of 4.5 to 24.9% [13]. However, these studies were conducted in three to 20 gyms [14, 15], involving 117 to 510 individuals [15, 16], and thus may not be representative of the populations of these cities.

In the current study, we examined AS use in the population of resistance training practitioners because this population includes both current and future users of AS. Characterizing these individuals is important because information on the profile of those who have already used, is currently using, or intends to use AS can contribute to the creation and improvement of public policies aimed at preventing abusive use of AS. Thus, this study aimed to verify the prevalence and profile of users and non-users of AS among resistance training practitioners.

## Methods

This study was approved by the Ethics Committee of the Pontifical Catholic University of Paraná (PUCPR), (CAAE1.524.203/2016) and was conducted according to the Consolidated Standards of Reporting Trials Statement and Helsinki Declaration of 1975 revised in 2000. All persons gave their written informed consent (Additional file 1) prior to their inclusion in the study. Details that might disclose the identity of the subjects under study were omitted.

### Sample

The survey was conducted in the city of Curitiba-PR (Brazil), which has approximately 1.9 million inhabitants [17]. The number of gyms and their locations were obtained from the Regional Council of Physical Education of Paraná (CREF-PR). A total of 680 fitness centers (May/2016) were identified, including resistance training centers (gyms). Only resistance training centers were included, resulting in a total of 286 centers. The number of resistance training centers was calculated with a confidence interval of 95%, and assuming that *p* = q = 50%, we calculated a total of 100 resistance training centers, with an error of 7.9%. These 100 centers were used to estimate the population of resistance training practitioners in the city. Gyms were selected in a systematic randomized manner and proportional to the number of gyms in the 10 administrative regions of the city, the geographical criterion of which is determined by the Institute of Research and Urban Planning of Curitiba [18]. Personal contact was made with those responsible for the gyms to explain the purpose of the study and to obtain authorization.

In these gyms, individuals of both sexes aged above 18 years and enrolled in resistance training in different periods (morning, afternoon, and night) were identified. An average of 481 practitioners of resistance training per gym was identified. The total number of individuals was calculated with a sampling error of 1.25% and confidence interval of 95%, resulting in a total of 5884 individuals. These individuals were selected in proportion to the number of practitioners of resistance training in each gym. In total, 5773 questionnaires were distributed from December 2016 to May 2017, with a sample error of 1.26%.

### Instruments for data collection

We prepared self-administered questionnaire (Additional file 2) containing 32 questions based on several articles in the literature [5, 19, 20]. The questions dealt with gender, age, profession, marital status, schooling, socioeconomic classification [21], practice time of resistance training, duration and objective of the training, nutritional monitoring, use of supplements, and use of AS. The questionnaire was developed and validated through the clarity, construct and content indices. Health professionals validated the aspects of construction and content, while the clarity aspect was validated with individuals of the same class, age and lifestyle of the individuals who would be researched. A pilot study was conducted that the questionnaire could be used with the intended population.

The questionnaire was constructed using the application KoboCollect (KoboCollect, Cambridge, Massachusetts, United States) in a Samsung Tablet Model Tab 2 (Samsung, Campinas, São Paulo, Brazil).

### Procedure of data collection

A pre-training with researchers was conducted to standardize the approach and application of the questionnaire. The pre-training was followed by a pilot study in the PUCPR gym involving 30 individuals. These data were not included in the survey.

Data collection was performed throughout the day: 25–30% of the questionnaires were distributed in the morning, another 25–30% in the afternoon, and the remaining 40–50% in the evening until the expected number of questionnaires per gym was reached. When needed, the researchers returned to the gym at another day.

The researchers were in uniform, positioned at the entrance of the gym, and identified. The subjects were approached at the beginning or end of the workout, and given explanation of the purpose of the research. Those who accepted to participate in the study signed an informed consent form. The researchers explained the filling out of the questionnaire and clarified any possible doubts. The subjects were left alone so that they could fill the questionnaire without any influence.

### Data analysis

The data were transferred from the application KoboCollect to an Excel sheet (Microsoft, Redmond, Washington, United States) and to the IBM SPSS 20.0 software (IBM SPSS, Armonk, New York, United States). Initially, we performed exploratory descriptive analysis of frequency distribution and percentages, with the results presented in tables. Normality of data distribution was analyzed by the Komolgorov-Smirnov test. Statistical significance was analyzed using the chi-square test and z-test, and indicated by *p* < 0.05.

For a better understanding of the results, the sample was separated into four groups: the non-user (Gnu), former user (Gex), current user (Gus), and future user (Gfu) groups.

We also calculated the prevalence ratio of AS users and non-users according to gender and age. For this calculation, the generalized Poisson regression model was used (*p* < 0.05). Only for this last calculation, the Gex and Gus groups were merged, forming a new group of AS users, whereas the groups Gfu and Gnu were merged to form a new group of non-AS users.

## Results

In total, 5773 individuals from 100 gyms participated in the study. Among these participants, 83.2% did not use, 9.1% formerly used, 3.4% currently used, and 4.3% intended to use AS. The prevalence of AS use in the Gex and Gus groups was 16.9 and 6.5% among men and women, respectively.

The average age of the subjects was lower for men (31.3 ± 10.4 years) than for women (33.2 ± 11.3 years) (*p* = 0.001). Table 1 presents the profile of the participants. There was a higher percentage of men (57.1%) than women (42.9%). The Gex, Gus, and Gfu groups showed higher percentages of men than that in the Gnu group. In the Poisson model, the prevalence ratio was as follows: for each female user of AS, there was 2.6 male users of AS (*p* = 0.001).

**Table 1:** Profile of the study participants

Variables	Total *n* (%)	Gnu *n* (%)	Gex *n* (%)	Gus *n* (%)	Gfu *n* (%)	p
Sex						
Male	3297 (57.1%)	2546_a_ (53.0%)	400_b_ (76.2%)	159_b_ (82.0%)	192_b_ (76.8%)	0.0001
Female	2476 (42.9%)	2258_a_ (47.0%)	125_b_ (23.8%)	35_b_ (18.0%)	58_b_ (23.2%)	
Age						
18 to 29 years	2836 (49.1%)	2291_a_ (47.7%)	258_a.b_ (49.1%)	109_b_ (56.2%)	178_c_ (71.2%)	0.0001
30 to 44 years	2108 (36.5%)	1725_a_ (35.9%)	245_b_ (46.7%)	74_a_ (38.1%)	64_c_ (25.6%)	
45 to 59 years	722 (12.5%)	682_a_ (14.2%)	22_b_ (4.2%)	11_b_ (5.7%)	7_b_ (2.8%)	
≥ 60 years	107 (1.9%)	106_a_ (2.2%)	0_b_ (0%)	0_b_ (0%)	1_a.b_ (0.4%)	
Marital Status						
Single	3296 (57.1%)	2654_a_ (55.2%)	327_b_ (62.3%)	115_a.b_ (59.3%)	200_c_ (80.0%)	0.0001
Married	2131 (36.9%)	1839_a_ (38.3%)	180_a_ (34.3%)	69_a_ (35.6%)	43_b_ (17.2%)	
Divorced	306 (5.3%)	272_a_ (5.7%)	17_b_ (3.2%)	10_a.b_ (5.2%)	7_a_._b_ (2.8%)	
Widow	40 (0.7%)	39_a_ (0.8%)	1_a_ (0.2%)	0_a_ (0.0%)	0_a_ (0.0%)	
Schooling						
CHE	2865 (49.6%)	2416_a_ (50.3%)	269_a_ (51.2%)	99_a_ (51.0%)	81_b_ (32.4%)	0.0001
IHE	1156 (20.0%)	917_a_ (19.1%)	116_a_ (22.1%)	47_a.b_ (24.2%)	76_b_ (30.4%)	
CHS	1270 (22.0%)	1066_a_ (22.2%)	94_b_ (17.9%)	32_a.b_ (16.5%)	78_c_ (31.2%)	
IHS	212 (3.7%)	180_a_ (3.7%)	16_a_ (3.0%)	4_a_ (2.1%)	12_a_ (4.8%)	
CBE	182 (3.2%)	153_a_ (3.2%)	19_a_ (3.6%)	7_a_ (3.6%)	3_a_ (1.2%)	
IBE	88 (1.5%)	72_a.b_ (1.5%)	11_b_ (2.1%)	5_b_ (2.6%)	0_a_ (0.0%)	
Socioeconomic Class						
Class A	1732 (30%)	1429_a_ (29.7%)	165_a.b_ (31.4%)	74_b_ (38.1%)	64_a_ (25.6%)	0.0001
Class B1	1285 (22.3%)	1080_a_ (22.5%)	112_a.b_ (21.3%)	31_b_ (16.0%)	62_a_ (24.8%)	
Class B2	1769 (30.6%)	1472_a.b_ (30.6%)	147_b_ (28.0%)	62_a.b_ (32.0%)	88_a_ (35.2%)	
Class C1	750 (13.0%)	638_a_ (13.3%)	67_a.b_ (12.8%)	15_b_ (7.7%)	30_a.b_ (12.0%)	
Class C2	190 (3.3%)	159_a_ (3.3%)	20_a_ (3.8%)	7_a_ (3.6%)	4_a_ (1.6%)	
Class D-E	47 (0.8%)	26_a_ (0.5%)	14_b_ (2.7%)	5_b_ (2.6%)	2_a.b_ (0.8%)	

*CHE* Complete higher education, *IHE* Incomplete higher education, *CHS* Complete high school, *IHS* Incomplete high school, *CBE* Complete basic education, *IBE* Incomplete basic education. Class A: upper class. Class B1: upper middle class. Class B2: middle class. Class C1: lower middle class. Class C2: lower class. Class D-E: no working. Z-test for proportions. Different letters in the row indicate significant differences (*p* < 0.05). Chi square test for *p* value

There were higher percentages of individuals aged between 18 and 29 years in the Gfu group, between 30 and 44 years in the Gex group, and between 45 and 59 years in the Gnu group. In the Poisson model, for each participant aged 45–59 years in the Gex and Gus groups, there were 2.9 participants aged 30–44 years and 2.4 participants aged 18–29 years (*p* = 0.001).

More than 50% of the individuals were single, with higher percentages of single participants in the Gfu and Gex groups than in the Gnu group. On the contrary, the percentage of married participants was the lowest in the Gfu group, and the percentage of divorced participants was lower in the Gex group.

Most of the participants completed upper secondary education, and the percentage of these participants was the lowest in the Gfu group. The highest percentage of individuals with incomplete upper and lower secondary education was shown in the Gfu group.

The socioeconomic classification A (upper class) and B2 (middle class) showed the highest percentages of resistance training practitioners, with higher percentage of class A in the Gus group than in the Gnu group. The percentages of class B1 (upper middle class) and C1 (lower middle class) were lower in the Gus group than in the Gnu group. The percentage of class D/E (no working) was higher in the Gex and Gus groups than in the Gnu group. This socioeconomic classification is based in 35.

Table 2 shows percentages of AS users according to training characteristics. The percentage of participants who practiced resistance training up to 6 months and between 6 months and 1 year was higher in the Gnu group than in the other groups. However, the percentage of participants who practiced between 6 months and 1 year was already increasing in the Gfu group, equivalent to that in the Gnu group. The percentage of participants who practiced between one and 3 years was the highest in the Gfu group. The percentage of those who practiced for more than 3 years was higher in the Gex, Gus, and Gfu groups.

**Table 2:** Percentage of use or non-use of anabolic steroids according to training characteristics

Variables	Total *n* (%)	Gnu *n* (%)	Gex *n* (%)	Gus *n* (%)	Gfu *n* (%)	p
Duration of bodybuilding						
< 6 months	1298 (22.5%)	1246_a_ (25.9%)	21_b_ (4.0%)	6_b_ (3.1%)	25_c_ (10.0%)	0.0001
≥ 6 months and < 1 year	821 (14.2%)	753_a_ (15.7%)	24_b_ (4.6%)	6_b_ (3.1%)	38_a_ (15.2%)	
≥ 1 year and < 3 years	1328 (23.0)	1139_a_ (23.7%)	70_b_ (13.3%)	34_b_ (17.5%)	85_c_ (34.0%)	
≥ 3 years	2326 (40.3%)	1666_a_ (34.7%)	410_b_ (78.1%)	148_b_ (76.3%)	102_c_ (40.8%)	
Frequency per week						
2 times	437 (7.6%)	420_a_ (8.7%)	8_b_ (1.5%)	2_b_ (1.0%)	7_b_ (2.8%)	0.0001
3 times	1237 (21.4%)	1163_a_ (24.2%)	44_b_ (8.4%)	6_c_ (3.1%)	24_b_ (9.6%)	
4 times	1526 (26.4)	1328_a_ (27.6%)	121_b_ (23.0%)	22_c_ (11.3%)	55_a.b_ (22.0%)	
5 or more times	2573 (44.6%)	1893_a_ (39.4%)	352_b_ (67.0%)	164_c_ (84.5%)	164_b_ (65.6%)	
Objective						
Hypertrophy	2953 (51.2%)	2177_a_ (45.3%)	398_b_ (75.8%)	172_c_ (88.7%)	206_c_ (82.4%)	0.0001
Weight loss	2326 (40.3%)	2082_a_ (43.3%)	132_b.c_ (25.1%)	40_c_ (20.6%)	72_b_ (28.8%)	0.0001
Resistance	2183 (37.8%)	1972_a_ (41.0%)	133_b_ (25.3%)	29_c_ (14.9%)	49_b.c_ (19.6%)	0.0001
Strength	2001 (34.7%)	1673_a_ (34.8%)	179_a_ (34.1%)	65_a_ (33.5%)	84_a_ (33.6%)	0.946
Other	474 (8.2%)	423_a_ (8.8%)	41_a_ (7.8%)	5_b_ (2.6%)	5_b_ (2.0%)	0.0001
Practicing another activity						
Yes	3161 (54.8%)	2699_a_ (56.2%)	272_a_ (51.8%)	80_b_ (41.2%)	110_b_ (44.0%)	0.0001
No	2612 (45.2%)	2105_a_ (43.8%)	253_a_ (48.2%)	114_b_ (58.8%)	140_b_ (56.0%)	

z-test for proportions. a,b,c: different letters in the row indicate significant differences (*p* < 0.05). Chi square test for *p* value

The percentage of those who trained two to three times a week was higher in the Gnu group than in the other groups. The same was shown by the percentage of those who trained four times a week, but the percentage in the Gfu group increased. The percentage of those who trained five or more times per week was higher in the Gex, Gus, and Gfu groups.

The Gex, Gus, and Gfu groups showed higher percentages of muscle hypertrophy, which is one of the main goals of resistance training practitioners. Moreover, the percentages of weight loss and endurance were lower in these groups than in the Gnu group. The percentage of individuals who practiced other activities besides resistance training was higher than that of individuals who did not practice other activities, and lower in the Gus and Gfu groups than in the Gex and Gus groups. The most practiced activity was running (32.4%), followed by soccer (22.5%), cycling (14.6%), fights (13.5%), and dance and swimming (5.7%). The percentage of practitioners of fights was higher in the Gex, Gus, and Gfu groups than in the Gnu group (*p* = 0.0001). The percentages of other activities were not different between the groups.

In addition, the number of individuals in the afternoon was higher in the Gex (41.3%), Gus (46.4%), and Gfu (42.8%) groups than in the Gnu group (35.4%) (*p* = 0.001).

Table 3 presents the percentage of use of anabolic steroids according to nutritional monitoring and use of supplements. It was observed that most of the resistance training practitioners did not have nutritional monitoring and did not use supplements. The percentage of participants who had a nutritionist and used supplements was higher in the Gex, Gus, and Gfu groups.

**Table 3:** Percentage of use of anabolic steroids according to monitoring by a nutritionist and use of supplements

Variables	Total *n* (%)	Gnu *n* (%)	Gex *n* (%)	Gus *n* (%)	Gfu *n* (%)	p
Nutritionist						
Yes	1654 (28.7%)	1243_a_ (25.9%)	203_b_ (38.7%)	119_c_ (61.3%)	89_b_ (35.6%)	0.0001
No	4119 (71.3%)	3561_a_ (74.1%)	322_b_ (61.3%)	75_c_ (38.7%)	161_b_ (64.4%)	
Supplements						
Yes	2334 (40.4%)	1612_a_ (33.6%)	384_b_ (73.1%)	174_c_ (89.7%)	164_d_ (65.5%)	0.0001
No	3439 (59.6%)	3192_a_ (66.4%)	141_b_ (26.9%)	20_c_ (10.3%)	86_d_ (34.4%)	
Type						
Protein	2143 (91.8%)	1455_a_ (90.3%)	364_b_ (94.8%)	169_b_ (97.1%)	155_a.b_ (94.5%)	0.0001
Amino Acid	807 (34.6%)	446_a_ (27.7%)	180_b_ (46.9%)	104_c_ (59.8%)	77_b_ (47.0%)	0.0001
Maltodextrin	463 (19.8%)	259_a_ (16.1%)	94_b_ (24.5%)	68_c_ (39.1%)	42_b_ (25.6%)	0.0001
Pre-workout supplement	801 (34.3%)	466_a_ (28.9%)	164_b_ (42.7%)	98_c_ (56.3%)	73_b_ (44.5%)	0.0001
Other	346 (14.8%)	200_a_ (12.4%)	69_b_ (18.0%)	40_b_ (23.0%)	37_b_ (22.6%)	0.0001

Z-test for proportions. a,b,c: different letters in the row indicate significant differences (*p* < 0.05). Chi square test for *p* value

Protein (whey protein) was the most consumed supplement among resistance training practitioners, followed by amino acids and pre-workout supplements. The percentage of individuals consuming amino acid, maltodextrin, pre-workout supplement, and other supplement was higher in the Gex, Gus, and Gfu groups than in the Gnu group, and the percentage of individuals using protein supplement was higher in the Gex and Gus groups than in the Gnu group. The other supplements used were omega 3, vitamins, creatine, thermogenics, caffeine, hypercaloric, glutamine, albumin, and post-workout supplement.

Among those who underwent nutritional monitoring, there was a higher percentage of individuals who used supplements (42.3%) than those who did not use supplements (19.4%) (*p* = 0.0001).

## Discussion

In this study it, can be observed that most of the participants did not use AS, whereas 9.1% formerly used, 3.4% currently used, and 4.3% thought intended to use AS. Previous studies [19, 20] evaluating AS use among resistance training practitioners grouped individuals who had used AS together with those who were using AS, thus resulting in lower prevalence than that found in our study; if the prevalence in the Gex and Gus groups were grouped, the prevalence would be higher (12.5%) than that in the previous study.

The sample size of gyms and participants in the literature also varied. A study in Germany carried out roughly 15 year ago evaluated 113 gyms and 621 individuals, and showed a prevalence of AS use of 13.5% [19]. In Stockholm, Sweden, the prevalence was 3.8% with 64 gyms and 1746 individuals [22]. In Al-Ain, United Arab Emirates, the prevalence was 22.1% with 18 gyms and 154 individuals [23]. However, other studies were restricted to smaller samples; for example, a study in El Paso, United States, evaluated three gyms and 516 individuals, showing a prevalence of 11.0% [24]. Moreover, a study in Boston, United States, examined five gyms and 511 individuals, showing a prevalence of 5, 1% [7]. The variability of the prevalence among these studies can be attributed to not only the sample distribution, namely the numbers of gyms and individuals, but also the regional and own characteristics of the samples. For example, in the Netherlands, a study involving 92 gyms and 718 individuals obtained a prevalence of AS use of 1% [25]. However, compared with the other studies, this study had a higher percentage of women (64%) and higher mean age (43.4 ± 13.6 years), which may explain why the number of women using AS was shown lower than that of men using AS.

The percentage of individuals who formerly used AS in the present study (9.1%) was higher than that in a meta-analysis involving 271 articles (3.3%) [10]. However, in this meta-analysis, the sample was heterogeneous because it included adolescents, university students, prisoners, military, bodybuilders, athletes, and sedentary people, among others. On the contrary, the present study examined only resistance training practitioners.

In Brazil, a systematic review presented a prevalence range of AS use of 2.1 to 31.6%, with a heterogeneous sample [26]. Specifically, in resistance training practitioners, the prevalence ranged from 4.5 to 24.9%. These studies showed the profile of various regions in terms of AS use, but some had relatively small sample sizes of both gyms and individuals.

The distinction between former (Gex) and current (Gus) users helped us understand the profile of AS users. Some individuals may have used AS to experiment at some point in their life and had not used it any more, whereas others may use AS recurrently. In a study conducted in Sweden, was assessed individuals who used AS at some point in their life (2.6%), within less than 12 months (0.9%), and within less than 30 days (0.3%) [22]. The results showed that, as in the present study, the number of individuals who had used AS is higher than that of individuals currently using AS.

In the present study, it was possible to calculate prevalence ratio owing to the large sample size. There was a higher prevalence of AS users among men than among women, which corroborated with literature [7, 27]. Furthermore, other studies presented a prevalence close to 5.7% [28] and 7% [14]. However, some studies did not report the use of AS by women [15, 23, 29]. AS consumption is lower among women because they do not wish to become extremely muscular or develop male characteristics [30]. In comparison, among men, the motivation of using AS goes beyond developing the ideal body; they use AS to gain status, admiration, and popularity in their social environment [6]. In addition, AS usage allows acceptance from and identification with their peers [31].

The current study, questionnaires were distributed proportionally to the number of individuals per period. The afternoon period presented a greater percentage of former, current, and future users of AS; thus, it was concluded that the highest prevalence of AS use occurred in the afternoon. This finding was not observed in any other study. We hypothesized that the afternoon showed greater number of AS users because the gyms were emptier during this period than during other periods in the day, which minimized interference during the course of training because the devices were shared with other practitioners.

In the present study, participants aged between 18 and 29 years presented the highest percentage of future AS users. Therefore, this age group should be the target of future education and preventive actions regarding abusive AS use. Participants aged between 30 and 44 years old showed high prevalence of former AS users (11.6%), whereas in other studies, high prevalence was shown by those aged between 18 and 29 years [14–16].

The search for improvements in quality of life, along with increasing advancement of antiaging therapies, may have prompted the increase in the use of AS. Older people have shown interests in hormone replacement; however, this treatment may cause a number of undesirable effects in young people [32]. Age-related physiological decline and societal pressure on body image are factors contributing to the increased use of AS in older men [33].

In the age group of 45–59 years, the prevalence of non-users of AS was the highest. It was verified that the frequency of AS and the number of resistance training practitioners decrease with age, showing that this activity is probably not the most sought after by this age group. Among single participants, there were high percentages of former and future users of AS. Although this high prevalence of former users of AS is supported by the literature [16, 22, 34], no previous study evaluated individuals who intended to use AS.

Most of the participants had complete upper secondary education, as observed in other studies [13, 15]; thus, it was suggested that people with lower level of education showed lower adherence to resistance training. There was a higher percentage of individuals with incomplete upper and lower secondary education among future users of AS, corresponding to individuals aged between 18 and 29 years, which showed the highest frequency of future users of AS. This finding emphasized the need for orientation regarding the use of AS in schools and universities, considering that AS use was also high in individuals with incomplete upper secondary education [29].

The prevalence of AS use was higher in individuals who had been practicing resistance training for a longer time. Previous studies have shown that AS use is higher in those who have been training for more than 2 years [14, 19, 23]. The frequency of sessions per week also showed similar, in accordance with the literature, which showed higher prevalence for train five times or more per week [14, 16, 19]. This higher frequency may be related to the awareness of the need for concomitant use of AS with training to obtain the desired result. Moreover, considering that these individuals train regularly, another possible motivation may be that they have not achieved the desired result solely through training.

This study found that the main objective of resistance training was muscle hypertrophy, which is consistent with the literature [13, 33]. The percentage of individuals who formerly used, currently using, or intended to use AS was high among these practitioners because they sought higher increase in muscle mass [35].

The number of individuals who had nutritional monitoring (28.7%) in this study was higher than that found by Silva et al., 2007 [20] (13.9%). In our study, among these individuals, 42.3% used supplements, and this number is higher than the 26% observed by Pellegrini et al., 2017 [13]. The prescription of supplements is the responsibility of a nutritionist, but instead, the resistance training coaches gave the prescription [36].

The use of food supplements in Brazil has varied from 20.5 to 94.0% among practitioners of physical activities [13, 36, 37], and our current result was within this range (40.4%). Among AS users, these values increased to 80% [14], which is close to our current results, namely 73.1 and 89.7% in former and current users of AS, respectively. Thus, it was indicated that individuals who use AS are more likely to use supplements [13, 22]. In our results, protein was the most common supplement used, in accordance with previous studies [13, 14, 16], because protein supplements help to achieve muscle hypertrophy, which is one of the objectives of these practitioners [38]. Also, the addiction must be considered as a potential complication of innapropriate use of AS [39].

The limitations of the present study included possible response bias. The participants may have not provided a response representing reality because they were using AS for non-therapeutic reasons.

## Conclusion

The prevalence of former and current users of AS among resistance training practitioners was 9.1 and 3.4%, respectively, suggesting the need for actions to prevent abusive use of AS. The gym environment encouraged the use of AS owing to aesthetic appeal. However, the profile of AS users should be considered in the preventive actions.

The present study revealed that the prevalence of AS use was higher among single men in the afternoon than in other times of the day, and that the high prevalence of supplement use by users of AS. Besides resistance training, another activity that revealed a high prevalence among AS users was fighting. In addition, resistance training practitioners did not show interest in using AS at the beginning, but individuals who had been trained for longer had a higher prevalence of use. It was also observed that younger individuals between the ages of 18 and 29 with incomplete high school or higher education have a higher prevalence in relation to the use of AS. Thus, there is a need for orientation actions in schools and universities as well as the action of health professionals such as doctors, nutritionists, and physical educators to prevent abusive use of AS.

## Supplementary information

**Additional file 1.** Informed consent.

**Additional file 2.** Questionnaire with question about use of AS.

## Data Availability

All data generated or analysed during this study are included in this published article. The datasets generated and/or analysed during the current study are not publicly available due, because the confidentiality of the participants, but are available from the corresponding author on reasonable request.

## Abbreviations

AS: Anabolic steroids
CREF-PR: Regional Council of Physical Education of Paraná
Groups: the non-user (Gnu), former user (Gex), current user (Gus), and future user (Gfu) groups
HDI: Human development index
IPPUC: Institute of Research and Urban Planning of Curitiba
PUCPR: Pontifical Catholic University of Paraná
