# Supplementary material for: Prevalence and profile of users and non-users of anabolic steroids among resistance training practitioners
Source: BMC Public Health. 2019 Dec 9;19:1650. doi: 10.1186/s12889-019-8004-6 (PMC6902556; doi:10.1186/s12889-019-8004-6)
Supplement: Supplementary file 1 — Additional file 1. Informed consent. [file 12889_2019_8004_MOESM1_ESM.docx]

Informed Consent

You are being invited as a volunteer to participate in the study “Prevalence and profile of users and non-users of anabolic steroids among resistance training practitioners", which aims to verify the amount of anabolic users in the Curitiba city gyms, as well as the profile of these users. We believe it is important to identify the profile of them, especially related to the information they have about the use of anabolic steroids and the performance of tests that may indicate important changes in health.

PARTICIPATION IN THE STUDY

My participation in this study will be to complete a questionnaire about the use of AS.

RISKS AND BENEFITS

I was warned that from the research to be carried out, I can expect some benefits, such as: contributing to identify the prevalence of the use of AS in gyms in the city of Curitiba. I also received that it is possible that the following discomforts may happen, such as: feeling some discomfort when answering a question from the questionnaire because it is a controversial subject, which may expose my privacy. Attitudes must be taken to reduce it, such as clarifying the importance of the study and that I am not being judged for my actions, but my collaboration in the study can help to understand the issues surrounding the use of anabolic steroids in society.

CONFIDENTIALITY AND PRIVACY

I understand that my privacy will be respected. My name or any other data or information will be kept confidential. Researchers are responsible for the safekeeping and confidentiality of data, as well as non-exposure of research data.

AUTONOMY

Assistance is assured throughout the research, and I must have free access to all additional information about the study and its consequences; everything I want to know before, during and after my participation. I was also informed that I can refuse to participate in the study, or withdraw my consent at any time, without giving reasons.

RISK AND INDEMNITY

However, if I have any expenses arising from participating in the research, such as transportation, food, among others, there will be reimbursement by money.

CONTACT

The researchers involved in this project are Ericson Pereira (PUCPR) and Aline Cristina Batista Rodrigues Johann (PUCPR) and I will be able to contact them at (41) 3271-1561 or (41) 98730-7377.

The Human Research Ethics Committee is formed by a group of people who are working to ensure that yours rights as a research participant have been respected. It has an obligation to assess whether the research has been planned and is being carried out ethically. If you find that the research is not being conducted as you have imagined or is being harmed in any way, you may contact the PUCPR Research Ethics Committee at (41) 3271-2292 between Monday and Friday from 08h00 to 17h30 or by e-mail nep@pucpr.br.

DECLARATION

I declare that I have read and understood all the information contained in this Informed Consent and have had the opportunity to discuss the information in this term. All my questions have been answered and I am pleased with the answers. I understand that I will receive a signed and dated copy of this document and that another signed and dated copy will be filed by the researcher responsible for the study.

Finally, having been advised on the content of all of the foregoing and understanding the nature and purpose of the study, I express my free consent to participate, being fully aware that there is no economic value to be received or paid by my participation.

| **Name:** |  |
| --- | --- |
| **Phone number:** |  |
| **e-mail:** |  |

Date:________________________________________________________________________

_____________________________________ ______________________________

Participant signed Research signed
